# Paralytic ileus as first symptom of Miller Fisher syndrome: A case report

**DOI:** 10.1097/MD.0000000000030434

**Published:** 2022-09-09

**Authors:** Xiubin Liu, Xiqi Chen, Yongkun Zhou, Xiaoxia Zhang

**Affiliations:** a Shandong Institute of Literature and Culture, Shandong University of Traditional Chinese Medicine, Jinan City, Shandong Province, People’s Republic of China; b The First Clinical Medical College, Shandong University of Traditional Chinese Medicine, Jinan City, Shandong Province, People’s Republic of China; c Department of General Surgery, Affiliated Hospital of Shandong University of Traditional Chinese Medicine, Jinan City, Shandong Province, People’s Republic of China.

**Keywords:** case report, Miller Fisher syndrome, paralytic ileus

## Abstract

Miller Fisher syndrome (MFS), regarded by many scholars as a variant of Guillain Barre syndrome (GBS), accounts for approximately 5% to 10% of GBS cases. The typical clinical manifestations of MFS are extraocular muscle paralysis, ataxia, and tendon reflex loss or disappearance. To date, intestinal obstruction has rarely been reported as the initial symptom.

**Patient concerns:**

A 48-year-old woman presenting with abdominal pain and distention was diagnosed with paralytic ileus. There was no significant improvement in symptoms after symptomatic treatment. After that, the patient developed visual rotation, with limited binocular abduction and adduction, and ataxia. Anti-ganglioside testing revealed positive anti-ganglioside antibodies.

**Diagnosis:**

The patient was diagnosed as MFS.

**Interventions:**

The early stage is mainly symptomatic treatment of paralytic ileus. After MFS was diagnosed, the patient was given large amounts of immunoglobulin and hormone shock therapy.

**Outcomes:**

After 1 week, the symptoms of intestinal obstruction and MFS gradually improved. The patient was later discharged automatically for financial reasons. Six months after discharge, the patient was interviewed by telephone, and she had recovered.

**Conclusion:**

To date, intestinal obstruction has rarely been reported as the initial symptom. In case of inconsistencies between the imaging examinations and clinical symptoms, neuroelectrophysiology and cerebrospinal fluid puncture should be performed, striving for timely detection and treatment.

## 1. Introduction

Miller Fisher syndrome (MFS), regarded by many scholars as a variant of Guillain Barre syndrome (GBS),^[1,2]^ accounts for approximately 5% to 10% of GBS cases.^[3]^ The typical clinical manifestations of MFS are extraocular muscle paralysis, ataxia, and tendon reflex loss or disappearance. Other cases of patients exhibiting dysphagia, photophobia, taste loss,^[4]^ limb numbness, and facial paralysis^[5]^ have also been reported. To date, intestinal obstruction has rarely been reported as the initial symptom. It is also generally assumed that MFS involves only the peripheral nervous system and that the central nervous system is not involved. In this case, the fact that paralytic intestinal obstruction was the first symptom may be related to the fact that the nerves innervating the intestine were the earliest involved nerves.

## 2. Case presentation

A 48-year-old female was admitted to the hospital with abdominal pain and distension, accompanied by cessation fatigue and defecation for 2 weeks. At the time of admission, the patient also exhibited cough and expectoration, no nausea or vomiting, no dizziness or headache, and no blurred vision.

The patient had a cold, cough and no fever during the week before admission. She had self-administered the oral Chinese patent medicine Lianhua Qingwen Capsule for the cold. She had no history of chronic diseases such as hypertension or diabetes. She also had no history of fever or specific infections in the month before the disease and no family history of genetic predisposition. The patient denied consuming alcohol or having allergies to food or medicine.

**Figure 1.**
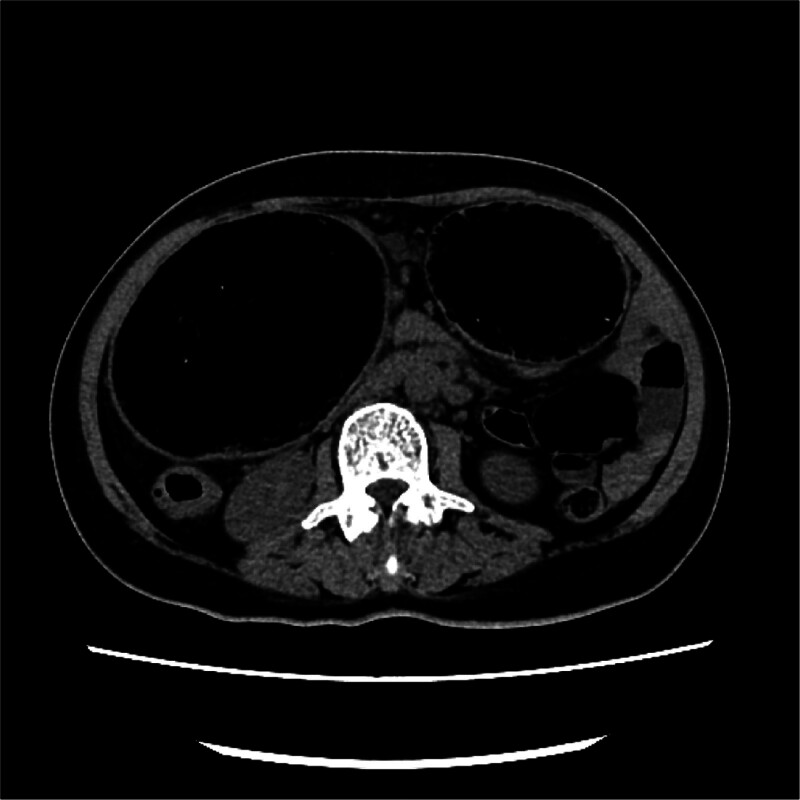
Full abdominal CT (Model: Toshiba 320-row Aquilion ONE, manufactured by Toshiba, country: Japan): transverse colon dilated, considered as incomplete intestinal obstruction.

The results of the physical examination that was performed upon admission were as follows: yellowish complexion, clear consciousness, lack of mood disturbances, equally sized and round bilateral pupils, light sensitivity, lack of obvious abnormalities in cardiopulmonary auscultation, abdominal distention, slight tightness and mild tenderness in the right abdomen and around the umbilicus, lack of liver and spleen abnormalities upon palpation, weakened bowel sounds, and normal muscle strength in both lower limbs. The patient’s laboratory test results are shown in Table 1. The patient’s imaging examination results showed intestinal obstruction (Fig. 1).

**Table 1:** Laboratory test results.

Blood tests	Result	Reference values
Red blood cells	3.56 × 10^12^/L	3.80–5.10 × 10^12^/L
Hemoglobin	100 g/L	115–150 g/L
Albumin	29.1 g/L	44.0–55.0 g/L
Potassium	3.79 mmol/L	3.50–5.30 mmol/L
Calcium	2.03 mmol/L	2.11–2.52 mmol/L
Sodium	138 mmol/L	137–147 mmol/L

**Figure 2.**
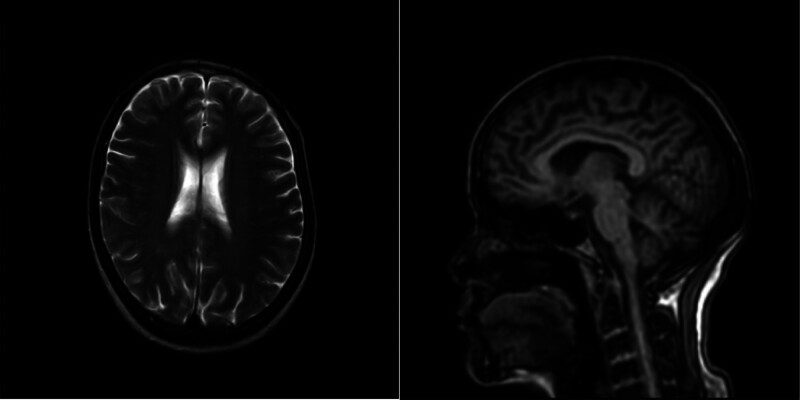
Brain MRI: bilateral cerebral hemispheres had a little ischemic degeneration.

After admission, the patient was treated with fasting, continuous gastrointestinal (GI) decompression, nutritional support, correction of electrolyte disorder, inhibition of GI gland secretion, and an enema of a hospital-made cleansing decoction of herbal medicine (the decoction is made of rhubarb, mirabilite, Immaturus aurantii and Magnolia officinalis). The patient’s intestinal obstruction symptoms were slightly improved after the treatment. Five days after admission, the patient had nausea and a choking cough with eating, accompanied by dizziness and rotation of the visual field. The results of the brain MRI are shown in Figure 2.

After symptomatic treatment, the patient’s symptoms did not significantly improve; visual objects were shadowed, there was binocular abduction, and adduction was limited. At that time, the results of the physical examination showed the following: clear consciousness, mood disturbances, clear and fluent speech, active and cooperative behavior during the physical examination, no enlargement or narrowing of the palpebral fissure, binocular abduction and limited adduction. There was vertical nystagmus when the eyes were looking upward. The muscle strength and muscle tension in the extremities were normal, and the tendon reflex in the extremities was weakened. The extremities were sensitive to both deep and shallow sensations, the patient exhibited ataxia, and no other pathological signs were present. The patient’s abdomen was distended, and there was tenderness around the umbilicus. Related laboratory test results are shown in Table 2.

**Table 2:** Laboratory test results.

Laboratory tests	Result
Anti-GM1 antibody	Positive
Anti-GQ1b IgG antibody	Positive
Anti- GT1a IgG antibody	Positive

GQ1b = anti-ganglioside.

Visual evoked potentials showed the following: bilateral P100 waveform differentiation was acceptable, repeatability was acceptable, and the incubation period was normal. Cerebrospinal fluid examination showed the following: protein-cell separation, an intracranial pressure of 200 mm Hg, protein levels of 190 mg/dL, a white blood cell count of 4 × 10^6^/L, normal glucose and chloride levels, and no obvious abnormalities upon electromyogram analysis. The patient was diagnosed with MFS.

Then, hormone shock therapy was administered as shown in Table 3. Human immunoglobulin C was injected intravenously at 27.5 g once a day (0.4 g/kg/d, patient weight 70 kg) for 5 consecutive days. At the same time, nutrition nerve adjuvant therapy was administered as follows: oral calcium carbonate 750 mg TID, intravenous potassium chloride 10 mL TID, oral famotidine 20 mg twice a day to prevent hormonal side effects, and oral vitamin B1 20 mg TID. The patient also received conventional treatment for paralytic ileus.

**Table 3:** Schedule of hormone shock therapy.

Drug name	Drug dose and frequency	Duration of medication	Mode of medication
Methylprednisolone	1000 mg QD	3d	IV
Methylprednisolone	500 mg QD	3d	IV
Methylprednisolone	250 mg QD	3d	IV
Methylprednisolone	120 mg QD	3d	IV
Prednisone	60 mg QD	Reduced by 5 mg per week and administration gradually stopped	PO

IV = intravenous injection, PO = peros, QD = once a day.

After 1 week, the symptoms of intestinal obstruction and MFS gradually improved. The patient was later discharged automatically for financial reasons. Six months after discharge, the patient was interviewed by telephone, and she had recovered.

## 3. Discussion

The initial diagnosis of MFS is based on the following core clinical symptoms: tendon reflex weakening or disappearance, ataxia, and extraocular muscle paralysis, which need to be confirmed by neurophysiological examination and cerebrospinal fluid analysis in the clinic.^[6]^ Currently, it is believed that 84% of MFS patients have a history of infection,^[7]^ and the pathogenesis is mainly related to autoimmunity after infection. The antibodies produced after infection directly attack the presynaptic membrane and synaptic gap sites, resulting in damage to peripheral nerve integrity and the loss of nerve conduction and other related functions.^[8]^ It is generally believed that more than 90% of MFS patients have anti-ganglioside antibodies, and the presence of anti-GD1b antibodies, anti-ganglioside-antibody and anti-ganglioside 3-immunoglobulin antibodies has also been reported.^[9]^ It is also generally assumed that MFS involves only the peripheral nervous system and that the central nervous system is not involved. However, in an in-depth study of MFS, it was found that the central nervous system can also be involved in the disease,^[10]^ and the brain stem and cerebellum are the most common sites of central nervous system involvement.^[11]^ The specific pathological mechanism remains unclear, and some scholars believe that it may be consistent with the previously believed idea that immune mediation leads to the destruction of the blood-cerebrospinal fluid barrier.^[12]^

The etiology of paralytic intestinal obstruction can involve a variety of diseases featuring dilated intestines and loss of intestinal peristalsis due to various causes. The common causes include the following: surgical stimulation; acute abdomen causing reflex paralytic ileus; electrolyte abnormalities; diseases such as intestinal obstruction; stimulation of abdominal inflammation; and intestinal nerve diseases.

The patient had no history of recent surgery, and there was no acute abdominal pain upon stimulation. Laboratory tests did not suggest severe infection and electrolyte abnormalities, and abdominal CT ruled out abdominal organ lesions. Intestinal vascular embolism was ruled out based on the patient’s symptoms, signs and CT examination. The patient had a history of previous infection, progressive extraocular muscle paralysis, ataxia, medulla oblongata paralysis, and respiratory depression, and the results of the laboratory tests were consistent with the diagnosis of MFS. The symptoms were relieved after large amounts of immunoglobulin and hormone shock therapy were administered. Therefore, it was inferred that MFS was the cause of the paralytic intestinal obstruction. There are currently few reports of cases of MFS that feature intestinal obstruction as the initial symptom, but there have been reports of GBS with intestinal obstruction as the initial symptom.^[13,14]^ Studies have shown that approximately two-thirds of GBS patients have symptoms of autonomic nerve damage.^[15]^ This includes blood pressure fluctuations, arrhythmias, vasomotor dysfunction, and GI motility dysregulation. GBS is caused by an infiltration of lymphocytes in the ganglion, hypothalamus and brainstem of the autonomic nervous system, which destroys the intramural ganglion cells, causing inflammation and edema of the autonomic ganglion and leading to the lysis of nerve cells.^[16]^ At the same time, some reports suggest that ganglioside autoantibodies, such as anti-GM1 antibodies,^[17]^ may be present, which can cause excitation disorders of the sympathetic and parasympathetic nervous systems, leading to hyperfunction or hypofunction of the sympathetic and parasympathetic nervous systems.^[18]^ The intimate supply of the GI tract with autonomic parasympathetic and sympathetic nerves can be targeted and then lead to intestinal obstruction. In this case, the fact that paralytic intestinal obstruction was the first symptom may be related to the fact that the nerves innervating the intestine were the earliest involved nerves.

## 4. Conclusion

Paralytic ileus is rarely reported as the first symptom of MFS. This disease can cause medulla oblongata paralysis and respiratory center inhibition, which is clinically very dangerous. Because of the lack of specificity in laboratory examination and imaging diagnosis methods, it is easy to miss the diagnosis in clinical practice. It is suggested that we improve the understanding of the disease, ask for a detailed medical history, and perform the physical examination more carefully. In the case of inconsistencies between the results of imaging examinations and the clinical symptoms, neuroelectrophysiology and cerebrospinal fluid puncture should be performed with the goal of performing timely detection and treatment.

## Author contributions

**Conceptualization:** Yongkun Zhou.

**Investigation:** Xiubin Liu, Xiqi Chen.

**Supervision:** Xiqi Chen, Xiaoxia Zhang.

**Writing – original draft:** Xiubin Liu, Xiqi Chen, Yongkun Zhou, Xiaoxia Zhang.

**Writing – review & editing:** Yongkun Zhou, Xiaoxia Zhang.

## References

[1] Wakerley BR Uncini A Yuki N. Guillain-Barré and Miller Fisher syndromes--new diagnostic classification. Nat Rev Neurol. 2014;10:537–44. 25072194 10.1038/nrneurol.2014.138

[2] Teener JW. Miller Fisher’s syndrome. Semin Neurol, 2012, 32:512–6. 23677659 10.1055/s-0033-1334470

[3] van den Berg B. Guillain-Barré syndrome: pathogenesis, diagnosis, treatment and prognosis. Nat Rev Neurol. 2014;10:469–82. 25023340 10.1038/nrneurol.2014.121

[4] Yagi Y Yokote H Watanabe Y. Taste impairment in Miller Fisher syndrome. Neurol Sci. 2015;36:809–10. 25116259 10.1007/s10072-014-1916-0

[5] Jung JH Oh EH Shin JH. Atypical clinical manifestations of Miller Fisher syndrome. Neurol Sci. 2019;40:67–73. 30232672 10.1007/s10072-018-3580-2

[6] Bukhari S Taboada J. A case of Miller Fisher syndrome and literature review. Cureus. 2017;9:e1048. 28367386 10.7759/cureus.1048 PMC5362277

[7] Tuzun E Kurtuneu M Lang B. Biekerstaft’s encephalitis and Miller Fisher syndrome associated with voltage gated potassium channel and novel antineuronal antibodies. Eur J Neurol. 2010;17:1304–7. 20236177 10.1111/j.1468-1331.2010.02993.x

[8] Rodella U Scorzeto M Duregotti E. An animal model of Miller Fisher syndrome: mitochondrial hydrogen peroxide is produced by the autoimmune attack of nerve terminals and activates Schwann cells. Neurobiol Dis. 2016;96:95–104. 27597525 10.1016/j.nbd.2016.09.005 PMC5102781

[9] Matsuzono K Ozawa T Yoshikawa K. A case report of Fisher syndrome with the detection of anti-GM3 and anti-GD1b IgG antibodies. Neurol Sci. 2019;40:891–3. 30471020 10.1007/s10072-018-3655-0

[10] Chikakiyo H Kunishige M Yoshino H. Delayed motor and sensory neuropathy in a patient with brainstem encephalitis. J Neurol Sci. 2005;234:105–8. 15936038 10.1016/j.jns.2005.02.011

[11] Teener JW. Miller Fisher’s syndrome. Semin Neurol. 2012;32:512–6. 23677659 10.1055/s-0033-1334470

[12] Segurado OG Krüger H Mertens HG. Clinical significance of serum and CSF findings in the Guillain-Barré syndrome and related disorders.. J Neurol. 1986;233:202–8. 3746360 10.1007/BF00314019

[13] Burns TM Lawn ND Low PA. Adynamic ileus in severe Guillain-Barré syndrome. Muscle Nerve. 2001;24:963–5. 11410925 10.1002/mus.1095

[14] Nowe T Hüttemann MDK Engelhorn T. Paralytic ileus as a presenting symptom of Guillain-Barré syndrome. J Neurol. 2008;255:756–7. 18283392 10.1007/s00415-008-0783-0

[15] Ropper AH Wijdicks EFM Truax BT. Guillain–Barré Syndrome. Contemporary Neurology Series. Philadelphia: FA Davis. 1991.

[16] Agrawal S Peake D Whitehouse WP. Management of children with Guillain-Barré syndrome. Arch Dis Child Educ Pract Ed. 2007;92:161–8. 18032711 10.1136/adc.2004.065706

[17] Ying H Han L Yuanhui X. Expression of GM1 antibody in patients with Guillain-Barre syndrome and its relationship with prognosis. J Contemporary Med. 2020;26:160–2.

[18] Patel MB Goyal SK Punnam SR. Guillain-Barré syndrome with asystole requiring permanent pacemaker: a case report. J Med Case Rep. 2009;3:5. 19126210 10.1186/1752-1947-3-5 PMC2628935

